# *In Vitro* Anti-Osteoporosis Properties of Diverse Korean *Drynariae rhizoma* Phenolic Extracts

**DOI:** 10.3390/nu6041737

**Published:** 2014-04-24

**Authors:** Suk-Nam Kang, Jong Seok Lee, Joung-Hyun Park, Jae-Hyeon Cho, Jae-Hong Park, Kwang-Keun Cho, Ok-Hwan Lee, Il-Suk Kim

**Affiliations:** 1 Department of Animal Resources, Daegu University, Gyeongsan 712-714, Korea; E-Mail: whitenightt@hanmail.net; 2 Department of Food Science and Biotechnology, Kangwon National University, Chuncheon 200-701, Korea; E-Mail: jongseoklee78@gmail.com; 3 Department of Food Science and Technology, Institute of Food Science, Pukyong National University, Busan 608-737, Korea; E-Mail: pdc327@pknu.ac.kr; 4 Institute of Life Science, College of Veterinary Medicine, Gyeongsang National University, Jinju 660-701, Korea; E-Mail: jaehcho@gsnu.ac.kr; 5 Department of Animal Resource and Science, Dankook University, Choongnam 330-714, Korea; E-Mail: atom1965@hanmail.net; 6 Department of Animal Resource Technology, Gyeongnam National University of Science and Technology, Jinju 660-758, Korea; E-Mail: chotwo2@gntech.ac.kr

**Keywords:** *Drynariae rhizoma*, extraction solvent, phenolic compounds, antioxidant, anti-osteoporosis

## Abstract

*Drynariae rhizoma* has been used to prevent bone loss that occurs with increasing age. However, the chemical compounds in extracts that act on bone metabolism in herbal medicine are poorly understood. This study aimed to investigate and compare the extraction efficacy of polyphenolic compounds, antioxidant activity, and *in vitro* anti-osteoporosis properties of water extract (DR-DW) and ethanol extract (DR-EtOH) from *D. rhizoma*. Total phenolics and flavonoids were better extracted with 70% EtOH, and this extraction method also resulted in higher antioxidant activity and *in vitro* anti-osteoporosis properties in these extracts. In particular, the contents of phloroglucinol, protocatechuic acid ethyl ester, 2-amino-3,4-dimethyl-benzoic acid, 3-(3,5-dimethyl-pyrazol-1-yl)-benzoic acid, chlorogenic acid, syringic acid, trans-ferulic acid, (−)-epigallocatechin, epigallocatechin gallate, quercetin dehydrate, luteolin and emodin in DR-EtOH were higher than those in DR-DW. These results indicated that DR-EtOH could be a good source of natural herbs with anti-osteoporosis properties.

## 1. Introduction

Osteoporosis is a leading cause of fractures, especially among the elderly [1]. Reactive oxygen species (ROS) are considered to be involved in the onset of a number of age-associated conditions [2,3]. The free radical theory of aging provides much support for the role of ROS, such as superoxide, hydrogen peroxide and hydroxyl radicals in the initiation and progression of the aging process [4]. Aged animals have been shown to produce higher levels of ROS compared with younger animals. In addition, increased oxidative damage to DNA, proteins and lipids has been reported in aged animals [5]. A deleterious effect of oxidative stress in bone and an increase in ROS with advancing age represents a pathophysiological mechanism underlying age-related bone loss (2). ROS produced by osteoclasts stimulates and facilitates an imbalance between bone formation and bone resorption in rodent bone [6,7]. Excessive oxidative stress may lead to extensive bone loss and skeletal fragility, characteristic of osteoporosis [8,9]. The process of bone formation, remodeling and healing involves a coordinated action of various cell types. Advances in understanding the biology of osteoblast cells during these processes have been enabled through the use of various *in vitro* culture models from different organs [10]. During the proliferative period, osteoblast cells undergo DNA synthesis and cell division, resulting in a rapid increase in cell number until confluent. At this point, osteoblasts produce alkaline phosphatase (ALP) that indicates the occurrence of mature osteoblasts [11,12].

Several medications such as estrogen, bisphosphonates, calcitonin, calcium products, ipriflavone and anabolic steroids have been reported to be effective for curing osteoporosis [13,14,15]. However, these medications may have serious side effects, may not improve bone quality, or may not reduce susceptibility to fracture [16,17]. Recently, oriental traditional medicines have been re-evaluated by clinicians because these medicines have fewer side effects and are more suitable for long-term use compared with chemically synthesized medicines [18,19]. *Drynariae rhizoma* has been known as one of the most important edible herbs used in Korean and Chinese medicine. This plant has great pharmacological effect on inflammation, hyperlipemia, arteriosclerosis and osteoporosis diseases [20,21,22]. However, little phytochemical investigation has been conducted into the phenolic metabolites and biological activities of *D. rhizoma*. Plant polyphenols present in the human diet are of great interest as they possess potential antioxidant activity and anti-osteoporosis properties in their function as free radical scavengers [23]. The methodology used to analyze phenolic compounds in edible herbs generally includes extractions with solvents, such as ethanol, methanol, acetone or mixtures of these with water [24]. Different extraction methods can be applied to obtain *D. rhizoma* extracts. The variety of extraction conditions used (type of solvent, concentration, time, and temperature) can potentially affect the polyphenolic profile of the extracts, thus making comparisons between studies difficult [25].

In this study, we investigated the effects of water and ethanol extracts from *D. rhizoma* on polyphenolic compound contents, antioxidant activity, and proliferation and differentiation of cultured mouse osteoblastic cells *in vitro*.

## 2. Materials and Methods

### 2.1. Preparation of Drynariae Rhizoma Extracts

*D. rhizoma* whole plants purchased from a market in Korea were washed with distilled water, dried in an oven at 45 °C for 48 h, ground to powder with an electrical blender and stored at room temperature in hermetically sealed plastic bags prior to extraction. Powdered *D. rhizoma* (100 g) was extracted with distilled water and 70% ethanol (each 1000 mL) in a flask under thermal reflux for 24 h and then filtered. The extraction was repeated three times. The extracts were filtered through Whatman No. 2 filter paper (Whatman Ltd., Maidstone, UK), concentrated with a vacuum evaporator at 40 °C and completely lyophilized in a freeze drier (PVTFD10R, ILSIN Bio Co., Kyonggi, Korea). The yield of extraction was calculated and extract stored at −20 °C until use.

### 2.2. Extraction and Purification of Phenolic Acids and Flavonoids

*D. rhizoma* extract (0.1 g) was placed in a screw-cap vial wrapped with aluminum foil and extracted with 50 mL of acidified 50% methanol (formic acid, pH 2.39). The mixture was vortexed for 30 s and flushed with nitrogen for 2 min. The vial was allowed to stand with intermittent shaking (200 rpm) at room temperature for 60 min. Then, the extract was centrifuged at 4000 rpm for 10 min at 4 °C and the supernatant was collected. The pellet was subjected to a second extraction of 15 min with 50 mL of solvent. The supernatants were combined and evaporated in a rotary evaporator at 40 °C. The residue was dissolved in 10 mL of solvent containing 0.01 mg/mL *p*-coumarin and filtered through a 0.45 μm membrane filter, and 10 μL was injected for HPLC–DAD analysis.

### 2.3. Reagents, Standards, and Solvents

Solvents for the HPLC-DAD analyses were of analytical grade and were purchased from Sigma (St. Louis, MO, USA). Water was purified by a Milli-Q Plus system from Millipore (Milford, MA, USA). The following commercial compounds were used as standards: Gallic acid, pyrogallol, 4-hydroxy benzhydrazide, epigallocatechin, vanillic acid, (+)-catechin hydrate, syringic acid, phloroglucinol, *p*-anisic acid, dexamethasone, chlorogenic acid, caffeic acid, *p*-coumaric acid, trans-ferulic acid, naringin, 2-amino-3,4-dimethyl benzoic acid, coumarin, morin hydrate, luteolin, hesperetin, alizarin, biotin, *trans*-chalcone, rutin hydrate, myricetin, quercetin, rhein, 3-hydroxyflavone, and emodin were purchased from Sigma–Aldrich Co. Catechin gallate and protocatechuic acid were obtained from Santa Cruz Biotech (Santa Cruz, CA, USA). All other chemicals were analytical grade.

### 2.4. HPLC-DAD Analysis

HPLC-DAD analyses were performed on an Agilent series 1100 HPLC instrument allowing the determination at different wavelengths (280, 320, and 370 nm) of single molecules belonging to different subclasses. In detail, the Nucleosil 100-5 C-18 column (250 mm × 4.0 mm i.d., 5 μm particle size, Macherey-Nagel, Bethlehem, PA, USA) which was protected by a 10 mm guard column was used. The eluents were water at pH 3.29 by formic acid (0.035%, v/v) (A) and acetonitrile (B). The analyses were performed by a multistep linear solvent gradient as follows: 0–25 min, gradient 0%–15% B; 25–35 min, isocratic 30% B; 35–50 min, isocratic 40% B; 50–55 min, isocratic 100% B; 55–63 min, isocratic 50% B; 63–73 min, isocratic 0% B. The flow rate was 1.0 mL/min and oven temperature was 30 °C. The single compounds were quantified with HPLC-DAD using a five-point regression curve constructed with the standards. Calibration curves with *R*^2^ > 0.95 were used. The concentrations of gallic acid, pyrogallol, 4-hydroxy benzhydrazide, epigallocatechin, vanillic acid, (+)-catechin hydrate, syringic acid, phloroglucinol, catechin gallate, protocatechuic acid, *p*-anisic acid, 4-(3,5-dimethyl-pyrazol-1-yl) benzoic acid, and dexamethasone were calculated at 280 nm using each reference compound. The concentrations of chlorogenic acid, caffeic acid, *p*-coumaric acid, *trans*-ferulic acid, naringin, 2-amino-3,4-dimethyl benzoic acid, coumarin, morin hydrate (flavonol), luteolin, hesperetin, alizarin, biotin, *trans*-chalcone were evaluated at 320 nm using each external standard. The concentrations of rutin hydrate, myricetin, quercetin, rhein, 3-hydroxyflavone, and emodin were calculated at 370 nm using the reference compound. All of samples were analyzed in duplicate. Phenolic acids and flavonoids were identified by comparison with pure standards, isolated compounds, UV spectra, and relative retention time.

### 2.5. Superoxide Anion (O^2−^) Radical Scavenging Activity

Superoxide radicals were generated by the method described previously with a slight modification [26]. Samples (0, 10, 100, 1000 and 5000 μg/mL) were added to the reaction solution that contained 100 μL of 30 mM EDTA (pH 7.4), 10 μL of 30 mM hypoxanthine in 50 mM NaOH, and 200 μL of 1.42 mM nitro blue tetrazolium (NBT). The solution was preincubated at room temperature for 3 min, then 100 μL of 0.5 U/mL xanthine oxidase were added to the mixture and the volume was brought up to 3 mL with 50 mM phosphate buffer (pH 7.4). The solution was incubated at room temperature for 20 min, and then the absorbance was measured at 560 nm. The reaction mixture without xanthine oxidase was used as a blank (A1). The samples (A2) were added to the reaction mixture, in which O^2−^ was scavenged, thereby inhibiting the NBT reduction. Absorbance was measured and the decrease in O^2−^ was represented by A2-A1. Superoxide anion radical scavenging activity (SRSA) was calculated by the following equation: SRSA% = [(A2 − A1)/A1] × 100.

### 2.6. Hydroxyl Radical (OH^−^) Scavenging Activity

The scavenging activity of the *D. rhizoma* extracts on the hydroxyl radical (OH**^−^**) was measured by the deoxyribose method with some modifications [27]. The deoxyribose assay was performed in a 10 mM phosphate buffer (pH 7.4) containing 2.5 mM deoxyribose, 1.5 mM H_2_O_2_, 100 μM FeCl_3_, 104 μM EDTA and the *D. rhizoma* extracts (0, 10, 100, 1000 and 5000 μg/mL). The reaction was started by adding ascorbic acid to the final concentration of 100 μM. The reaction mixture was incubated at 37 °C for 1 h in the water bath. After incubation, the color was developed by adding 0.5% thiobarbituric acid followed by ice-cold 2.8% trichloroacetic acid in 25 mM NaOH and heating to 80 °C for 30 min. The *D. rhizoma* extracts (A2) were cooled on ice, and the absorbance was measured at 532 nm. The reaction mixture without the test sample was used as a control (A1). The hydroxyl radical scavenging activity (HRSA) was calculated by the following equation: HRSA% = [(A1 − A2)/A1] × 100.

### 2.7. Free Radical Scavenging Activity on DPPH Assay

The free radical scavenging activity of the *D. rhizoma* extracts (0, 10, 100, 1000 and 5000 μg/mL) was measured based on the method described with some modifications [28]. A DPPH solution (0.4 mM) in anhydrous ethanol was stirred for 30 min, and the absorbance of the solution was adjusted to 1.0 ± 0.1 at 490 nm. Then, 0.2 mL of the sample (or a control) was mixed with 0.8 mL of DPPH solution and incubated for 10 min in the dark at room temperature. The decrease in absorbance was measured at 490 nm. l-ascorbic acid was used as a positive control. The radical scavenging activity was calculated and expressed as a percentage using the following formula: DPPH radical scavenging activity% = [1 − (Atest/Bcontrol)] × 100.

### 2.8. Cell Culture

Osteoblastic cells derived from newborn KP100 CD-1 mouse calvaria (Oriental Bio Inc., Seoul, Korea) were isolated and cultured in plastic dishes containing αMEM (ICN pharmaceuticals, Inc., Aurora, OH, USA) supplemented with 10% fetal bovine serum (FBS) (HyClone, Logan, UT, USA), and 100 U/mL penicillin/streptomycin (GIBCO, Life Technologies, Roskilde, Denmark) in a CO_2_ incubator at 37 °C [20]. To measure cell growth, DNA synthesis and alkaline phosphatase activity, after the cells reached confluence, they were cultured in a proliferation medium (αMEM containing 0.5% FBS and 100 U/mL penicillin/streptomycin) for 48 h in the presence of water and ethanol extracts from *D. rhizoma* (0.1, 1, 10, 50 and 100 μg/mL). The water and ethanol extracts from *D. rhizoma* were dissolved in phosphate-buffered saline (PBS) and 20% dimethyl sulfoxide (DMSO), respectively.

### 2.9. MTT Assay

To evaluate the viability of osteoblastic cells, 3-(4,5-dimethylthiazol-2yl-)-2,5-diphenyl tetrazolium bromide (MTT) from Sigma (St. Louis, MO, USA) was used. Five mg/mL MTT solution was added into each well of 96-well culture plates, and the cells were then further incubated at 37 °C for 4 h. To measure the absorbance, 100 μL of acidic isopropanol were added to each well and the incubated solutions in the plates were read at 560 nm in a Microplate ELISA reader (Multiskan Ex, Thermo Scientific, Waltham, MA, USA).

### 2.10. ^3^H-TdR Assay

The levels of DNA synthesis resulting from the incubation of mouse osteoblastic cells with *D. rhizoma* extracts at different concentrations were measured. The cells were labeled with 1 μ Ci of [methyl-^3^H] thymidine (Amersham Pharmacia Biotech Inc., Buckinghamshire, UK) for the last 12 h of incubation and were then rinsed with PBS and collected with a cell harvester (Inotech Inc., Basel, Switzerland). The cell lysate was placed in a liquid scintillation counter (Packard Instrument Co., Downers Grove, IL, USA).

### 2.11. Alkaline Phosphatase (ALP) Activity

The activity of ALP was measured by the method described in [29]. Briefly, after various treatments, an aliquot of the supernatant was used to determine ALP activity by measuring the release of *p*-nitrophenol from *p*-nitrophenylphosphate solution at 30 °C for up to 5 min. The absorbance of *p*-nitrophenol product formed as a result of ALP reaction on *p*-nitrophenylphosphate was measured at 405 nm in a Microplate ELISA reader, and compared with serially diluted standards [12]. The activity of the enzyme is expressed as millimoles of *p*-nitrophenol per mg protein.

### 2.12. Statistical Analysis

The data from at least three experimental replicates were analyzed using ANOVA and Duncan’s multiple range tests. Statistical significance was accepted at a level of *p* < 0.05 (SAS-Institute, Cary, NC, USA, 1988). The results are shown as the mean values and standard deviations.

## 3. Results and Discussion

### 3.1. Extraction Yield, Total Phenolic and Flavonoid Contents

The yield of the water extract (9.58 ± 0.34%) was significantly higher (*p* < 0.01) than that of the ethanol extract (7.65 ± 0.52%) (data not shown). Total phenolic and flavonoid contents of the water extract (DR-DW) and the ethanol extract (DR-EtOH) obtained from *D. rhizoma* are shown in Table 1. The total phenolic and flavonoid contents of the ethanol extract (1583 mg/100 g and 9543 mg/100 g, respectively) were higher than those of the water extract (1323 mg/100 g and 7909 mg/100 g). Phytochemical investigation indicated the presence of complicated phenolic mixtures in the extracts from *D. rhizoma*. The contents of phloroglucinol, protocatechuic acid ethyl ester, 2-amino-3,4-dimethyl-benzoic acid, 3-(3,5-dimethyl-pyrazol-1-yl)-benzoic acid, chlorogenic acid, syringic acid, *trans*-ferulic acid, (−)-epigallocatechin, epigallocatechin gallate, quercetin dehydrate, luteolin and emodin in DR-EtOH were higher than those of DR-DW. In particular, phloroglucinol was detected in DR-EtOH, but not in DR-DW. Phloroglucinol and its derivatives, a major class of secondary metabolites, may be an effective treatment for diabetic complications [30]. *Trans*-ferulic acid is one of the most abundant phenolic acids, with biochemical and pharmacological activities widely present in plants [31,32]. (−)-Epigallocatechin is a flavan-3-ol, a type of chemical compound including catechin. It is one of the antioxidant chemicals found in food [33]. Therefore, these results suggest that the extraction solvent influences the polyphenolic profile from *D. rhizoma* and the polyphenol-enriched ethanol extract from *D. rhizoma* may be more effective on antioxidant activity.

**Table 1:** Total phenolic and flavonoid contents (dry basis) of water and ethanol extracts from *Drynariae rhizoma*.

Phenolic Compound	RT (min)	DR-DW (mg/100 g Extract)	DR-EtOH (mg/100 g Extract)
**Phenolic acid**			
*benzoic acid*			
phloroglucinol	7.27	-	129.29 ± 0.69
4-hydroxy benzhydrazide derivative	7.57	72.73 ± 0.05	72.63 ± 0.19
gallic acid	8.45	20.70 ± 0.42 ^a^	15.59 ± 0.29 ^b^
vanillic acid	21.91	32.18 ± 0.31 ^b^	42.94 ± 0.37 ^a^
protocatechuic acid ethyl ester	34.92	17.17 ± 19.84 ^b^	32.37 ± 0.18 ^a^
2-amino-3,4-dimethyl-benzoic acid	33.37	182.55 ± 0.63	206.44 ± 0.48 ^a^
*p*-anisic acid	34.62	9.41 ± 0.12 ^a^	2.94 ± 0.09 ^b^
alizarin	44.00	7.86 ± 0.06 ^a^	0.05 ± 0.07 ^b^
4-(3,5-dimethyl-pyrazol-1-yl)-benzoic acid	39.85	59.85 ± 0.06 ^b^	127.23 ± 0.17 ^a^
*cinnamic acid*			
chlorogenic acid	23.17	71.26 ± 0.36 ^b^	212.76 ± 1.76 ^a^
caffeic acid	23.16	6.43 ± 0.25 ^a^	5.91 ± 0.9 ^b^
syringic acid	24.10	4.09 ± 0.13 ^b^	27.37 ± 0.21 ^a^
*p*-coumaric acid	30.76	22.77 ± 0.01 ^a^	16.46 ± 0.05 ^b^
chlorogenic derivative	31.23	268.66 ± 0.18 ^a^	71.16 ± 0.85 ^b^
*trans*-ferulic acid	32.51	547.54 ± 0.62 ^b^	620.02 ± 0.03 ^a^
coumarin	35.66	-	-
**Total of phenolic acid**		1323.22 ± 19.85	1583.16 ± 0.46
*Flavonoids*			
*Flavanols*			
(+)-catechin hydrate	21.24	255.58 ± 0.59	258.41 ± 0.15
catechin gallate	33.77	13.38 ± 0.12	13.33 ± 0.19
gallocatechin	17.68	-	-
(−)-epigallocatechin	18.58	7,152.94 ± 2.91 ^b^	8,954.97 ± 1.46 ^a^
epicatechin	25.60	-	-
epigallocatechin gallate	27.27	42.18 ± 0.12 ^b^	70.18 ± 0.11 ^a^
quercetin hydrate	35.45	1.25 ± 0.07	1.40 ± 0.14
myricetin	37.59	-	-
morin hydrate	38.96	58.41 ± 14.23 ^a^	40.37 ± 0.19 ^b^
quercetin dihydrate	40.50	56.99 ± 0.02 ^b^	78.40 ± 0.35
morin derivative	42.19	10.23 ± 0.24	10.13 ± 0.10
kaempferol	42.43	7.26 ± 0.19	7.19 ± 0.09
3-hydroxyflavone	45.80	210.63 ± 0.23 ^a^	- ^b^
*Flavones*			
rutin hydrate	33.77	9.84 ± 0.20	9.74 ± 0.34
luteolin	40.67	7.08 ± 0.12 ^b^	18.48 ± 0.39 ^a^
*Flavanones*			
naringin	34.77	78.84 ± 0.20 ^a^	58.79 ± 0.13 ^b^
*Anthraquinones*			
rhein	44.47	-	-
emodin	45.83	4.48 ± 0.03 ^b^	22.07 ± 0.04 ^a^
**Total of flavonoids**		7909.11 ± 10.84 ^b^	9543.45 ± 1.07 ^a^

DR-DW, water extracts from *D. rhizoma*; DR-EtOH, 70% ethanol extracts from *D. rhizome*; the data presented as the mean ± SD of triplicate determinations; -, not detected; ^a,b^ Means ± SD were significantly different within the same row (*p* < 0.05).

### 3.2. Antioxidant Activity of D. rhizoma

ROS have been proposed to be causative in aging overall and may influence the severity of osteoporosis [2,4]. ROS such as superoxide are involved in bone resorption and bone degradation [6,7]. Evidence suggests that ROS leading to oxidative stress in bone can increase bone loss and bone weakness, which are characteristic of osteoporosis [3,8,9]. The phenolic contents of extracts from medicinal plants were very closely correlated with antioxidant activities [34].

**Figure 1:**
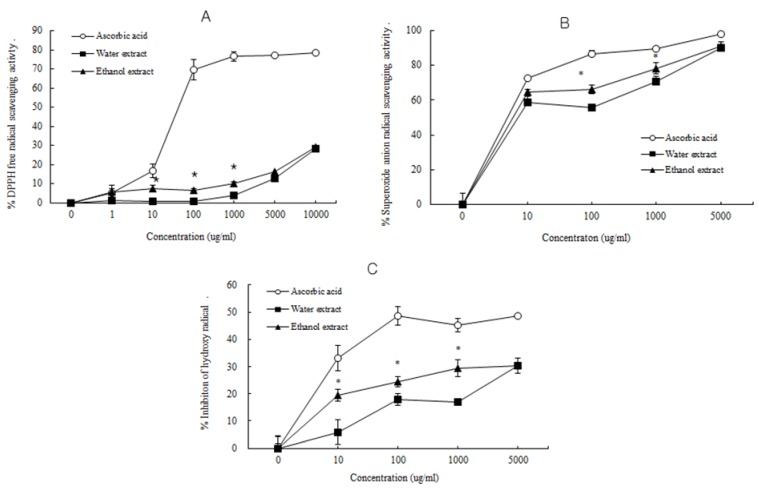
The radical scavenging activity of water and ethanol extracts from *Drynariae rhizoma*. (**A**) Free radical scavenging activity; (**B**) Superoxide anion radical scavenging activity; (**C**) Hydroxyl radical scavenging activity.

Hence, the antioxidant activities such as superoxide anion radical-, hydroxyl radical-, and DPPH radical-scavenging activities of water and ethanol extracts from *D. rhizoma* were tested. The free radical scavenging activity, superoxide anion (O^2−^) radical scavenging activity and hydroxyl (OH^−^) radical scavenging activity of water and ethanol extracts from *D. rhizoma* are shown in Figure 1. The ethanol extract from *D. rhizoma* exhibited significantly higher free radical scavenging activity (6.55% and 9.82%, respectively) and superoxide anion radical scavenging activity (66.29 and 78.31%, respectively) than those of the water extract (0.60% and 3.87%; 55.56% and 70.54%, respectively) at 100 and 1000 μg/mL concentrations. The ethanol extract from *D. rhizoma* exhibited significantly higher hydroxyl (OH^−^) radical scavenging activity (19.43%, 24.47 and 29.51%) than that of the water extract (5.91%, 17.81% and 17.71%) at 10, 100 and 1000 μg/mL concentrations, respectively. The higher antioxidant properties of the ethanol extract expressed as high superoxide anion radical scavenging activity and free radical scavenging activity compared with that of the water extract might be due to the high content of phenolic compounds (Table 1). Different solvents still yield different extract composition. The extraction yield and the antioxidant activity of the extracts from plants highly depend on the solvent polarity, which determines both qualitatively and quantitatively the extracted antioxidant compounds [35]. Water and ethanol are those most widely used because of their low toxicity and high extraction yield, with the advantage of modulating the polarity of the solvent by using ethanol/water mixture at different ratios [36]. Even if the highest extraction yields are achieved with water, the main drawback of the aqueous extraction is the low yield in antioxidants with low polarity or liposoluble antioxidants.

Solvents with different polarity have significant effects on total phenolic contents, extracted components, and antioxidant activities. Solubility of polyphenols and flavonoids depend on mainly on the hydroxyl groups and the molecular size and the length of hydrocarbon [37]. Numerous studies have reported some beneficial effects associated with the antioxidant activity and total phenols of plant extracts. Because oxidative stress is an important mediator of bone loss, administration of antioxidants might protect bones from osteoporosis and also might help in the acceleration of healing of fractured bones [38,39]. It was reported that *D. rhizoma* can protect osteoblasts against oxidative stress induced-damage [40,41]. Therefore, these results suggest that the ethanol extract from *D. rhizoma* might be more effective for oxidative stress-induced disease processes in aging bone compared with the water extract.

### 3.3. Influence *of* D. rhizoma on the Proliferation of Mouse Osteoblastic Cells

**Figure 2:**
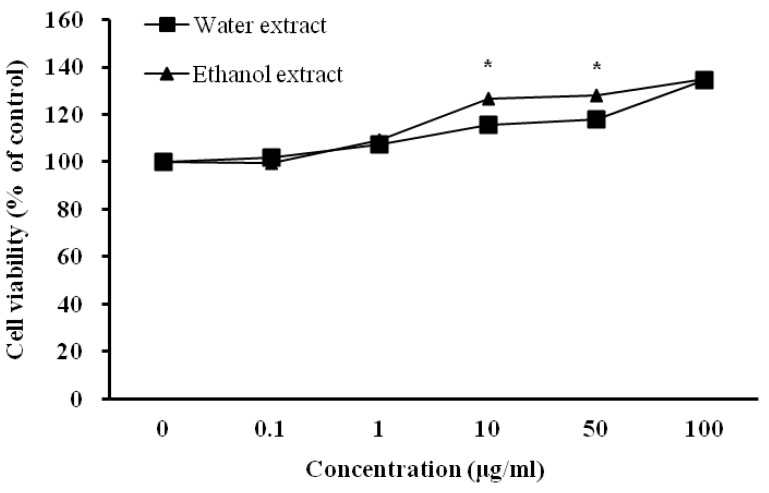
Effects of water and ethanol extracts from *Drynariae rhizoma* on the proliferation of mouse osteoblastic cells. Cells were treated with vehicle (excipient) or various concentrations of *D. rhizoma* extracts for 48 h. Results are expressed as percentage of control (vehicle). Significant differences were compared with vehicle control (*p* < 0.05).

**Figure 3:**
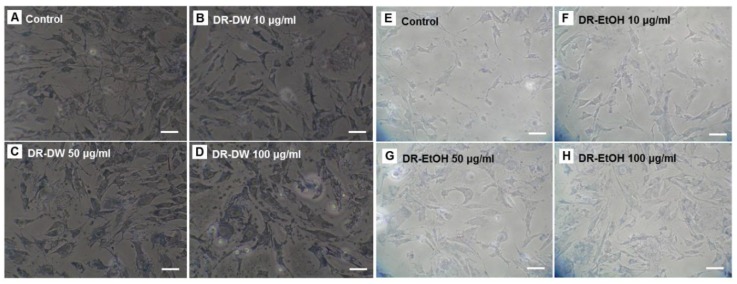
Morphological changes of mouse osteoblastic cells after treatment with various concentrations of DR-DW (A–D) and DR-EtOH (E–H) from *Drynariae rhizoma*. Scale bar = 20 μm.

The effect of *D. rhizoma* on the viability of mouse osteoblastic cells was measured using the MTT assay, which relies on the ability of viable cells to metabolically reduce the tetrazolium salt MTT to a purple formazan product, which can be quantified colorimetrically [42]. The water and ethanol extracts from *D. rhizoma* did not indicate any reduction in cell viability (Figure 2). In addition, both *D. rhizoma* extracts increased the cell viability of mouse osteoblastic cells (Figure 2). The ethanol extract was shown to have slightly higher cell viability than that of water extract at 10 and 50 μg/mL (*p* < 0.05). In a morphology study, the growth of mouse osteoblastic cells after 48 h of culture was affected by different concentrations of water and ethanol extracts from *D. rhizoma* as observed in different proliferation properties under a light microscope. In the control condition, the cells had a high density and a well-developed inter-cellular collagen network (Figure 3). However, when water and ethanol extracts were added to the medium at 10, 50 and 100 μg/mL, cell density of mouse osteoblastic cells was increased in a concentration-dependent manner.

**Figure 4:**
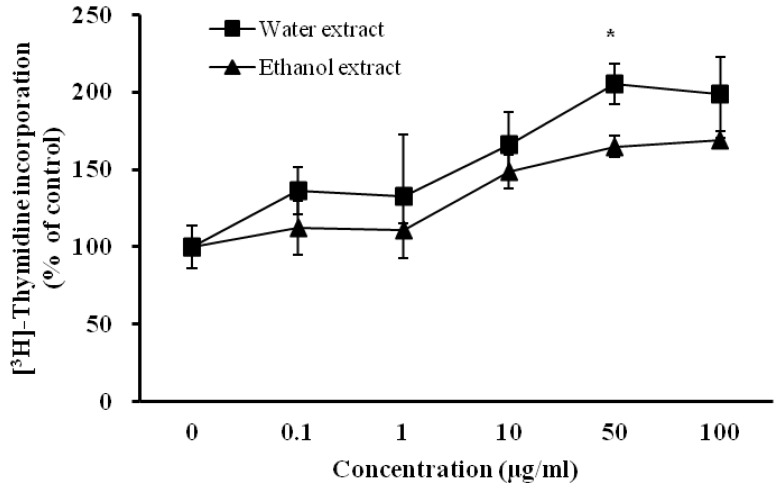
Effects of water and ethanol extracts from *Drynariae rhizoma* on DNA synthesis of mouse osteoblastic cells. Cells were treated with vehicle or various concentrations of *D. rhizoma* extracts for 48 h. Results are expressed as percentage of control (vehicle). Significant differences were compared with vehicle control (*p* < 0.05).

In addition, over-extended matrix networks were observed in media that contained 100 μg/mL of *D. rhizoma* extract (Figure 3). Cell growth was most evident at the highest concentration (100 μg/mL) in both extracts (Figure 2 and Figure 3). However, we did not find a morphology difference between water extract and ethanol extract-treated cells. To investigate whether *D. rhizoma* extracts increased the proliferation of mouse osteoblastic cells, we investigated the effect of *D. rhizoma* extracts on the DNA synthesis of mouse osteoblastic cells after treatment with *D. rhizoma* extracts (Figure 4). Incorporation of titrated [methyl-^3^H] thymidine has been used as a tool to assess the rate of DNA synthesis in cell culture [43]. The dose-response curve showed an increase in DNA synthesis after treatment with *D. rhizoma* extracts. Interestingly, we observed that the water extract of *D. rhizoma* increased DNA synthesis (205.38% of basal value) compared with the ethanol extract (Figure 4). Therefore, these results suggest that the ethanol extract from *D. rhizoma* could stimulate the proliferation of mouse osteoblastic cells to a greater degree than the water extract.

### 3.4. Influence of D. rhizoma on the Differentiation of Mouse Osteoblastic Cells

**Figure 5:**
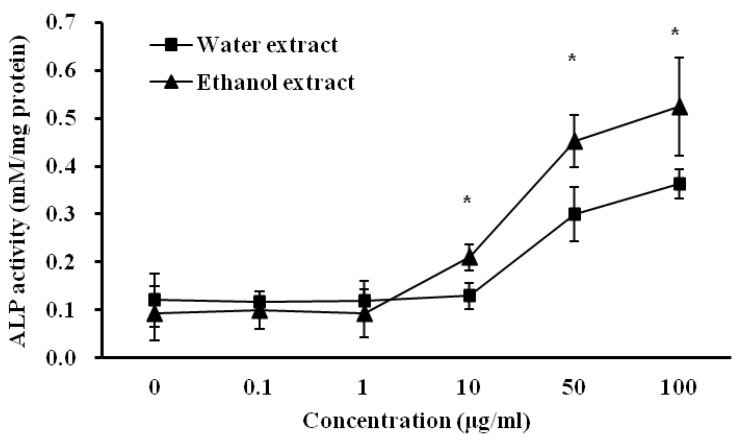
Effects of water and ethanol extracts from *Drynariae rhizoma* on the alkaline phosphatase of mouse osteoblastic cells. Cells were treated with vehicle or various concentrations of *D. rhizoma* extracts for 96 h. Each point represents the Mean ± SD. Significant differences were compared with vehicle control (*p* < 0.05).

ALP activity is the most widely recognized biochemical marker for osteoblastic activity. Although its precise mechanism of action is still poorly understood, this enzyme is believed to play a role in bone mineralization [11,12]. Figure 5 shows the effects of water and ethanol extracts from *D. rhizoma* on ALP activity in the proliferation medium after a 4-day culture period. The regression line for ALP is *y* = 0.0096 *x* + 0.3302, where the *y*-axis represents optical absorbance (OD) at 405 nm and the *x*-axis represents the activity of alkaline phosphatase (millimoles of *p*-nitrophenol per mg protein). The correlation coefficient of the standard curve was 0.9999 (data not shown). As the concentration of water and ethanol extracts increased to 50 and 100 μg/mL, the ALP activity of mouse osteoblastic cells increased (*p* < 0.05) to 2.48–3.00 times and 4.82–5.58 times, respectively, compared with the control activity. However, there were no significant differences in water and ethanol extracts compared with the control at lower (0.1 and 1 μg/mL) extract concentrations. The activities of the ethanol extract at 10, 50 and 100 μg/mL (0.21, 0.45 and 0.52 mM/mg protein, respectively) were slightly higher than those of the water extract (0.13, 0.30 and 0.36 mM/mg protein, respectively) (*p* < 0.05). In previous studies, *D. rhizoma* had stimulative effects on the proliferation and differentiation of various osteoblastic cells [11,12,20,21]. The inhibitory effects of genistein, a soybean isoflavone and vitamin d-containing plant extract on bone resorption are mediated through an estrogen-like action [44,45,46]. From quantitative and morphological observations on mouse osteoblastic cells, this study suggests that the ethanol extract from *D. rhizoma* may have a greater effect on the proliferation and differentiation of osteoblastic cells and activity of ALP compared with the water extract, suggesting that the ethanol extract from *D. rhizoma* might be more effective against disease processes in bone.

## 4. Conclusions

In conclusion, little research work has been published on the phenolic metabolites and biological activities of *D. rhizoma*. This study investigated the effects of water and ethanol extracts from *D. rhizoma* on polyphenolic compound contents, antioxidant activity, and proliferation and differentiation of cultured mouse osteoblastic cells *in vitro*. Although the study had limitations, these results indicated that the ethanol extract from *D. rhizoma* has higher content of polyphenolic compounds, antioxidant activity, and cell proliferation and differentiation activities of osteoblasts compared with the water extract. These results suggest that the ethanol extract of *D. rhizoma* might be more effective for disease control in aging bone.
